# Immunomodulatory Effect of Continuous Venovenous Hemofiltration during Sepsis: Preliminary Data

**DOI:** 10.1155/2013/108951

**Published:** 2013-07-23

**Authors:** Giuseppe Servillo, Maria Vargas, Antonio Pastore, Alfredo Procino, Michele Iannuzzi, Alfredo Capuano, Andrea Memoli, Eleonora Riccio, Bruno Memoli

**Affiliations:** ^1^Medical Intensive Care Unit, Department of Surgical and Anesthesiological Sciences, University Federico II of Naples, 80129 Naples, Italy; ^2^Department of Nephrology, University Federico II of Naples, 80129 Naples, Italy

## Abstract

*Introduction*. Severe sepsis and septic shock are the primary causes of multiple organ dysfunction syndrome (MODS), which is the most frequent cause of death in intensive care unit patients. Many pro- and anti-inflammatory mediators, such as interleukin-6 (IL-6), play a strategic role in septic syndrome. Continuous renal replacement therapy (CRRT) removes in a nonselective way pro- and anti-inflammatory mediators. *Objective*. To investigate the effects of continuous venovenous hemofiltration (CVVH) as an immunomodulatory treatment of sepsis in a prospective clinical study. *Methods*. High flux hemofiltration (Qf = 60 ml/Kg/hr) was performed for 72 hr in thirteen critically ill patients suffering from severe sepsis or septic shock with acute renal failure (ARF). IL-6 gene expression was measured by real-time PCR analysis on RNA extracted from peripheral blood mononuclear cell before beginning of treatment (T0) and after 12, 24, 48, and 72 hours (T1–4). *Results*. Real-time PCR analysis demonstrated in twelve patients IL-6 mRNA reduction after 12 hours of treatment and a progressive increase after 24, 48, and 72 hours. *Conclusions*. We suggest that an immunomodulatory effect might exist during CVVH performed in critically ill patients with severe sepsis and septic shock. Our data show that the transcriptional activity of IL-6 increases during CVVH.

## 1. Introduction 

 Sepsis is a great public health problem and represents the leading cause of death in intensive care units of developed countries. Different studies have reported a high population-based incidence of sepsis, and high percentages of intensive care unit (ICU) beds have been occupied by those patients [1–4].

 An increase of cytokines has been evidenced in plasma of patients presenting septic shock and has been associated with poor prognosis [5]. Microbial products are responsible for the induction of inflammation. This leads to local microvascular injury with potential dissemination and malignant sequelae at different organ levels. These effects are described as multiple organ failure (MOF). While there is a high concentration of proinflammatory mediators (IL-1, IL-6, IL-8, and TNF-*α*) at the site of infection, body's stress response contributes to an opposing systemic reaction by producing anti-inflammatory mediators (IL-4, IL-10, and IL-13). Often the production of proinflammatory cytokines is followed by secretion of inhibitors, such as soluble receptors or receptor antagonists. Bone et al. called this response “compensated antiinflammatory response syndrome” (CARS) [6]. Several lines of evidence suggest that compensated response and “systemic inflammatory response syndrome” (SIRS) may often coexist in the same patient but in different compartments [7]. In the “peak concentration hypothesis,” peaks of proinflammatory cytokines and peaks of anti-inflammatory cytokines coexist and the concept of cutting peaks, for example, through hemofiltration, may contribute to bring the patient to a nearly normal immunohomeostasis. The therapeutic approaches focused on decreasing cytokine production or neutralizing the effects of cytokines; continuous renal replacement therapy (CRRT) may play a role in the downregulation of the inflammatory response [8] by nonselective extracorporeal removal, mainly by absorption, of cytokines and other mediators, restoring the hemodynamic and the immunologic homeostasis. However, the hypothesis that hemofiltration can remove inflammatory mediators and its role in the sepsis therapy is still conflictual.

 A phase II clinical study showed no reduction of plasma levels of circulating IL-6, IL-8, IL-10, and TNF-*α* or occurrence of MOF in twelve patients with early septic shock or septic organ dysfunction who received 48 hours of continuous venovenous hemofiltration (CVVH) [9]. Ronco et al. estimated that CVVH with replacement fluid of 35 mL/Kg improves survival in critically ill patients with acute renal failure (ARF) admitted in two intensive care units (ICUs) [10]. However, it is still uncertain whether beneficial effect of high volume hemofiltration is the result of intensified blood purification or an effect on nonspecific cell-mediated immunity. Therefore, Dellinger et al. in the international guidelines for management of severe sepsis and septic shock recommended CVVH as the elective therapy for ARF in septic shock, allowing the management of fluid balance in hemodynamically unstable septic patients [11].

 Concentration of cytokines has been measured in many studies, but few assays sized neither the bioactivity of the cytokines nor their net effects on immune functions [12]. Circulating cytokines may just be the “tip of the iceberg,” implying that neither their presence nor their absence can reflect the complex interplay at the tissue level [13]. Despite the fact that their peak concentration may reflect an exacerbated production, these levels do not necessarily stand for enhanced bioactivity. To determine the balance of inflammatory response measurement of cytokines bioactivity may be superior to their absolute concentration. Moreover, variation of a single cytokine in the complex immune response syndrome could also reflect the change of other mediators. Therefore, we decided to measure interleukin-6 gene expression in mononuclear cells (PBMCs) harvested from septic patients, as a marker for immune function and proinflammatory bioactivity. Persisting high levels of IL-6, in fact, correlate with poor outcome in sepsis, and its concentration correlates with the concentration of other inflammation markers. 

 Aim of the present observational prospective study is to investigate the effect of high volume continuous hemofiltration on the transcriptional activity of PBMC, as a marker of immunomodulatory effect of this treatment in septic process. 

## 2. Materials and Methods 

### 2.1. Patients

The study was performed in the intensive care unit of the University of Naples “Federico II” after local ethics committee approval. Informed consent was obtained from patients' next of kin. From January 2007 to January 2008, we enrolled medical and surgical patients from our intensive care unit suffering from severe sepsis or septic shock with coexisting ARF, according to the American College of Chest Physicians/Society of Critical Care Medicine Consensus Conference criteria. Exclusion criteria were age >80 years, acute bleeding, and immunodepression.  Table 1 shows clinical characteristics of our patients. All patients received conventional intensive care therapies in accordance with the international sepsis guidelines [14], as intubation and mechanical ventilation with a tidal volume of 6 mL/kg and an upper limit plateau pressure   <30 cmH_2_O, sedation by continuous infusion according to clinical requirements, and intravenous antibiotics as indicated by microbiological resistance testing. All patients received inotropic support in addition to other vasoactive drugs, as clinically indicated, and fluid therapy by using crystalloids and colloids. Once the decision was made to proceed with renal replacement therapy, a double lumen catheter was inserted in femoral vein and continuous hemofiltration was started. 

### 2.2. Treatments

Ultrafiltration rate (Qf) was 4 l/hr. Blood flow rate was more than 200 mL/min. For ultrafiltration, we used a polyethersulfone filter (Acquamax HF12) with a surface area of 1.20 m^2^ and a maximal transmembrane pressure (TMP) of 600 mmHg. Anticoagulation was obtained with heparin 6.0 U/Kg/hr. In continuous venovenous hemofiltration, solute transport is achieved by pure convection. Solute flux across the membrane is proportional to the ultrafiltration rate (Qf) and the ratio between the concentration of solute in the ultrafiltrate and in the plasma water (sieving coefficient, *S*), since clearance is calculated from product Qf × *S*. When *S* is proximal to 1 (for solutes freely crossing the membrane), clearance is assumed to be Qf. Therefore, since ultrafiltration rate corresponds to clearance in continuous hemofiltration, it may be used as a surrogate of treatment dose. Two different machines were used for the study, both equipped with calibrated peristaltic blood pumps and fluid balance systems with calibrated scales. Replacement solution was added in the postdilutional mode. We used Accusol with potassium (Baxter Healthcare), which had a pH of 7.4, 35 mmol/L of bicarbonate, and 2 mmol/L of potassium, associated with other ionic compounds. Theoretical osmolarity of this solution was of 296 mOsm/L. Blood samples were obtained before the beginning of treatment (T0) and after 12, 24, 48, and 72 hours (T1–4). SAPS 3 [15, 16] and daily SOFA [17] were measured for each patient. 

### 2.3. IL-6 Gene Expression Analysis

 Blood samples (20 mL of heparinized blood) were collected from all patients to obtain plasma samples and isolate peripheral blood mononuclear cells (PBMCs). These cells were isolated by Ficoll-Hypaque (Flow Laboratories, Irwine, UK) gradient density centrifugation (400 ×g for 30 min). Then PBMCs were incubated in culture tubes (Falcon) in quantities of 3 × 106/mL and cultured for 24 h at 37°C in 5% CO_2_ saturated humidity incubator. After this step, cell-free supernatants were collected by centrifugation. PBMCs were pulverized with a blender and lysed using TRIzol reagent. Total RNA was extracted by the single-step method, using phenol and chloroform/isoamylalcohol. Four micrograms of total RNA were subjected to cDNA synthesis for 1 h at 37°C using the “Ready-To-Go You-Prime First-Strand Beads” Kit (Amersham Pharmacia Biotech Little Chalfont Buckinghamshire, UK) in a reaction containing 0.5 *μ*g oligo-dT (Amersham Pharmacia Biotech Little Chalfont Buckinghamshire, UK) [18].

Real-time quantitative PCR analysis for IL-6 gene was performed using ABI Prism 7500 (Applied Biosystem, Foster City, CA, USA) and the 5′ exonuclease assay (TaqMan technology). This assay uses a specific oligonucleotide probe, annealing between the two primer sites, which is labeled with a reporter fluorophore and a quencher. Cleavage of the probe by exonuclease activity of Taq polymerase during strand elongation releases the reporter from the probe resulting in an increase in reporter emission intensity owing to its separation from the quencher. This increment in net fluorescence is monitored in real time during PCR amplification. We have described the cDNA synthesis. Now, the cDNA was used for real-time PCR realized on 96-well optical reaction plates with cDNA equivalent to 20 ng RNA in a volume of 5 *μ*L reaction containing 1x TaqMan Universal MasterMix, optimized concentrations of FAM-labelled probe, and specific forward and reverse primer for the gene selected from Assay on Demand (Applied Biosystems Foster City, CA, USA). Controls included RNA subjected to RT-PCR without reverse transcriptase and PCR with water replacing cDNA. The results were analyzed using a comparative method, and the values were normalized and converted into fold change based on a doubling of PCR product in each PCR cycle, according to the manufacturer's guidelines.

### 2.4. Statistics

From Table 2, it can be deduced that results obtained from genetic expression of IL-6 are continuous variables function of times T0, T1, T2, T3, and T4. Such continuous variables are expressed as mean ± SD calculated with software “Statistica7” (Statsoft). Various results for the same genetic expression of IL-6 are confronted using ANOVA followed by Bonferroni, as post hoc test. Statistical significance is at the *P* < 0.05 level. 

## 3. Results 

**Figure 1:**
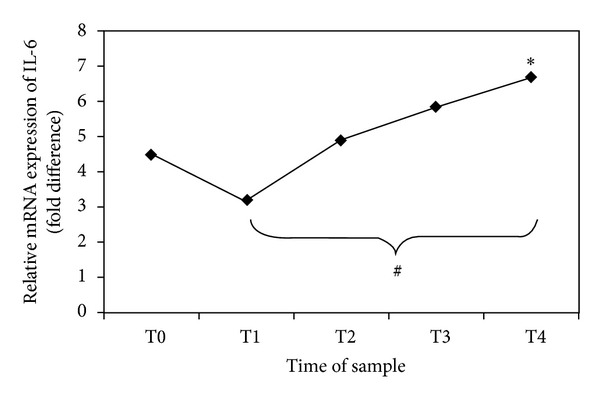
Gene expression of IL-6 in PBMCs obtained by real-time PCR analysis. The points show the relative expression of the mRNA abundance of IL-6. The values on the *y*-axis represent arbitrary units derived from the mean expression value for the gene of septic patients compared with the healthy subjects set at 1 (not reported in the figure). *P = 0.031 trend of relative mRNA expression of IL-6 (ANOVA). ^#^ *P* = 0.030 T1 versus T4 (Bonferroni post hoc correction).

**Figure 2:**
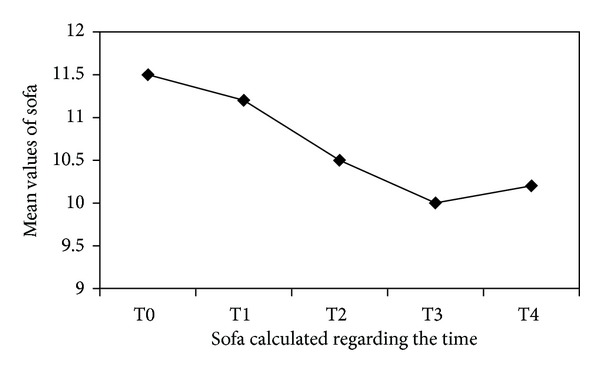
Mean values of daily SOFA measured for each patient. The points on *y*-axis represent that the mean values reach from this score during the time of treatment. The *x*-axis reported the different time of measurement.

 Results are expressed as number, percentage, or mean ± SD, as appropriate. Fifteen patients were considered eligible. Two patients developed severe hemodynamic instability during replacement therapy, and CVVH was discontinued. Therefore, thirteen patients were enrolled. The origin of sepsis was mostly medical but in some patients postsurgical and trauma related. Table 2 shows the results of real-time PCR analysis on blood samples and their mean values ± SD. In twelve out of thirteen patients, transcriptional activity behaviour is similar; that is, IL-6 mRNA is present in high amounts before filtration (T0), reduces after 12 hours of treatment, and progressively increases after 24, 48, and 72 hours. On the other hand, samples from patient number 4 show a different behaviour: transcriptional activity of IL-6 in this patient seems only to progressively reduce.  Figure 1 shows the trend of IL-6 gene expression variation in our thirteen patients (*P = 0.031) from T0 to T4 and a statistically significant increase between T1 versus T4 (*P* = 0.030).  Table 3 shows clinical data, SAPS 3, and daily SOFA for each patient.  Figure 2 shows trend of SOFA score modification in our thirteen patients.

## 4. Discussion

 Our prospective and clinical study, conducted on critically ill septic patients with ARF treated using high flux hemofiltration (Qf = 60 mL/Kg/hr) with a filter of polyethersulfone, has shown a progressive increase in transcriptional activity of IL-6 produced by PBMCs. In the early 90s, Bellomo et al. conducted a study on cytokines removal from circulation with dialysis [19]. The authors showed that CVVHD attenuated progression of ARF and removed inflammatory cytokines from blood of septic patients. After these results, some authors [9, 20, 21] hypothesized a role of CVVH not only as CRRT but also as immunomodulatory therapy for septic patients. In their “peak concentration hypothesis” [22], Ronco et al. suggest that the effect of nonspecific removal of unbound mediators and cytokines from blood compartment is a resetting to a lower level of immunedysregulation, both in prevalently proinflammatory and counterinflammatory phases of sepsis [10]. In their opinion, Ronco et al. affirmed that by eliminating the peaks, the system might restore its ability to achieve immune homeostasis [22]. More recently, Honoré et al. [20] shift their interest to the effect of high volume hemofiltration (HVHF) on the interstitium and tissue level. The authors conclude that both mediators and promediators are removed at interstitial and at tissue levels, secondary to removal from blood compartment, to a point where some pathways are completely shut down and cascades are blocked. At this point, called “Threshold point,” tissue damage and organ injury are stopped in their progression. Sander et al. [21] suggest that the entire amount of cytokines potentially eliminated through hemofiltration is probably lower when compared to endogenous production. Furthermore, hemofiltration membranes per se may increase cytokine production by activating mononuclear cells. In 2007, Horner et al. [23] concluded that plasma from septic patients contains substances mediating a substantially different immune response pattern compared with plasma from critically ill nonseptic patients. In an experimental study, Toft et al. [24] showed that infusion of endotoxin in pigs induced a significant increase in IL-6 and IL-10 levels in peripheral blood, but there was no difference in cytokine levels between CVVH-treated and nontreated septic animals. Thus, the author concluded that CVVH did not decrease plasma levels of cytokines, although it had been described that proinflammatory as well as anti-inflammatory cytokines are excreted in the ultrafiltrate. Moreover, De Vriese et al. [25] suggest that a decrease in concentration of cytokines in plasma was possible for a few hours after a change of filter, and in fact the filter rapidly became saturated with cytokines. 

Our prospective and clinical studies conducted on septic patients aim to evaluate the biological activity of leukocytes, as IL-6 production by PBMCs, during CVVH. We have applied to thirteen septic patients a CVVH with high flux (60 mL/Kg/hr), Qf of 4 L/hr, and polyethersulphone filter which allowed us to obtain a blood flow ≥ 200 mL/min. Our data show that transcriptional activity of an inflammatory cytokine such as IL-6 significantly increases during CVVH. In sepsis, however, impaired regulation may cause an excessive anti-inflammatory response, which generates monocyte “immunoparalysis” and exposes the host to further infections. Both processes (inflammation and anti-inflammation) are designed to act in response to specific stimuli in a well-balanced fashion defined as immunohomeostasis. Continuous therapies have been shown to provide clinical benefits beyond those expected by the simple substitution of renal function. In this view, it is possible to hypothesize that avoidance of peaks of mediator in blood may contribute to a partial restoration of the immunohomeostasis. When sepsis is viewed as a syndrome of immune suppression perspective, in which the immune effectors cells become dysfunctional and are no longer capable of guaranteeing a normal immune surveillance, we can hypothesize that increase of IL-6 transcriptional activity could be due to enhanced capability of immune cells to provide protection against external harmful stimuli. We therefore think that during CVVH, there might be a reactivation of immune capability of the leukocytes to produce inflammatory cytokines, restoring an immunologic balance.

In conclusion, we suggest that there might be an immunomodulatory effect of CVVH during sepsis. However, since response to sepsis should be viewed in a network perspective, within an array of interdependent mediators, it is important to further confirm this hypothesis. 

## Figures and Tables

**Table 1:** Characteristics of patients at baseline: gender, sex, underlying disease, origin of sepsis, and microbiology.

Patient	Gender	Age	Underlying disease	Origin of sepsis	Microbiology
N1	F	74	Gastric cancer, gastrectomy, and pulmonary thromboembolism	Abdominal	*Candida albicans *
N2	M	80	Left inferior pulmonary lobectomy, mesenteric infarction, and resection of small bowel	Abdominal	*Bacteroides fragilis *
N3	F	79	Pulmonary thromboembolism	Pulmonary	*Pseudomonas aeruginosa *
N4	F	60	Posttraumatic subdural haemorrhage	Pulmonary	*Acinetobacter baumannii *
N5	M	34	Multiple thoracic and abdominal trauma	Pulmonary	*Acinetobacter baumannii *
N6	F	51	Substitution of mitral valve after rheumatic fever cardiopathy	Mediastinal abscess	*Candida albicans Escherichia coli *
N7	M	56	Acute respiratory failure, lung cancer	Pulmonary	*Pseudomonas aeruginosa *
N8	M	55	Evacuation of cerebral haemorrhage	Pulmonary	*Klebsiella pneumoniae *
N9	M	51	Ligature of left external carotid, draining of facial, neck, and mediastinic abscess	Mediastinal abscess, infection of tracheostomy site	*Klebsiella pneumoniae Candida albicans *
N10	M	48	Multiple abdominal and pelvic trauma	Urinary	*Escherichia coli *
N11	F	59	Perforation of the colon, colonic resection, and peritonitis	Abdominal	*Escherichia coli, Enterobacter spp. *
N12	F	62	Acute respiratory failure, COPD	Pulmonary	*Pseudomonas aeruginosa Acinetobacter baumannii *
N13	M	46	Multiple thoracic abdominal and pelvic trauma	CVC	Staphylococcus coagulase negative

**Table 2:** IL-6 gene expression from real-time PCR analysis. Mean values. *P* values result of ANOVA followed by Bonferroni as post hoc test using the following matricesT1versus T0, T2 versus T0, T3 versus T0, and T4 versus T0.

Patient	T0	T1	T2	T3	T4
N1	3.5	1	3.6	5.15	6.7
N2	3	1.9	3.1	4.8	7
N3	4.6	2.8	3.9	5	6.2
N4	12	10.4	10	—	—
N5	9.8	8.5	10.3	11.6	—
N6	3.2	2.1	3.5	3.9	4.2
N7	1	1.8	—	—	—
N8	2.5	1	2.8	—	—
N9	3.1	2.4	2.7	5.8	9.3
N10	4.5	3	5.2	5.8	6.7
N11	4.2	2.9	5.1	6.2	6.8
N12	3.7	1.9	4.2	5.1	6.4
N13	3.9	2.2	4.5	5.4	6.9

Mean ± SD	4.53 ± 3.1	3.2 ± 2.8	4.91 ± 2.5	5.87 ± 2.1	6.68 ± 1.3
*P *		<0.05	>0.05	<0.05	<0.05

**Table 3:** SAPS 3 admission score, SOFA score calculated at 0, 12, 24, 48, and 72 hours, and outcome of each patient.

Patient	SAPS 3	SOFA 0	SOFA 1	SOFA 2	SOFA 3	SOFA 4	Outcome
N1	55 (26%)	6	6	7	7	8	Died
N2	27 (1%)	9	10	10	8	9	Died
N3	75 (66%)	11	9	9	11	10	Died
N4	56 (28%)	12	13	13	Stop CVVH	—	Died
N5	47 (13%)	15	12	12	13	Stop CVVH	Survived
N6	80 (74%)	16	18	12	16	18	Died
N7	68 (52%)	12	15	—	—	—	Died
N8	90 (85%)	13	15	15	—	—	Died
N9	57 (30%)	10	8	7	7	7	Survived
N10	52 (32%)	8	8	12	10	10	Survived
N11	64 (44%)	12	11	10	10	10	Survived
N12	67 (50%)	12	9	9	9	10	Survived
N13	56 (28%)	14	12	10	9	10	Survived

Mean ± SD	61.1 ± 15.6	11.5 ± 2.8	11.2 ± 3.4	10.5 ± 2.4	10 ± 2.8	10.2 ± 3.1	
